# Robust Least-SquareLocalization Based on Relative Angular Matrix in Wireless Sensor Networks

**DOI:** 10.3390/s19112627

**Published:** 2019-06-10

**Authors:** Yuyang Tian, Jing Lv, Shiwei Tian, Jinfei Zhu, Wei Lu

**Affiliations:** College of Communications Engineering, Army Engineering University of PLA, Nanjing 210042, China; constantin1994@163.com (Y.T.); jinglv1965@163.com (J.L.); zhujinfei1989@sina.com (J.Z.); kahn_lu@163.com (W.L.)

**Keywords:** wireless sensor networks, least-squares localization, convexity, relative angular matrix, non-line-of-sight

## Abstract

Accurate position information plays an important role in wireless sensor networks (WSN), and cooperative positioning based on cooperation among agents is a promising methodology of providing such information. Conventional cooperative positioning algorithms, such as least squares (LS), rely on approximate position estimates obtained from prior measurements. This paper explores the fundamental mechanism underlying the least squares algorithm’s sensitivity to the initial position selection and approaches to dealing with such sensitivity. This topic plays an essential role in cooperative positioning, as it determines whether a cooperative positioning algorithm can be implemented ubiquitously. In particular, a sufficient and unnecessary condition for the least squares cost function to be convex is found and proven. We then propose a robust algorithm for wireless sensor network positioning that transforms the cost function into a globally convex function by detecting the null space of the relative angle matrix when all the targets are located inside the convex polygon formed by its neighboring nodes. Furthermore, we advance one step further and improve the algorithm to apply it in both the time of arrival (TOA) and angle of arrival/time of arrival (AOA/TOA) scenarios. Finally, the performance of the proposed approach is quantified via simulations, and the results show that the proposed method has a high positioning accuracy and is robust in both line-of-sight (LOS) and non-line-of-sight (NLOS) positioning environments.

## 1. Introduction

In recent years, positioning and navigation technology has been playing an increasingly important role in many applications, such as public safety, law enforcement, rescue operations, traffic management, inventory tracking, home automation, etc. At the same time, location-based services also have significant commercial value [1]. The Global Navigation Satellite System (GNSS) is the most widely-used navigation and positioning technology, providing services that are suitable for most applications in an open environment [2]. However, the GNSS might fail to provide reliable services due to interference and in some challenging environments such as cities, forests and indoors, due to the weakness of GNSS signals.

An effective way of solving this problem is to supplement and enhance GNSS with terrestrial positioning systems. At present, there are several positioning systems, including cellular-based positioning, WiFi positioning, and ultra-wideband-based (UWB-based) positioning systems. In particular, with the development of large-scale multiple-input and multiple-output (MIMO) systems, positioning based on mmWave communication that is becoming an emerging research focus has also received increasing attention [3,4].

The sensors in the terrestrial system constitute a positioning network, and we are interested in locating the sensors based solely on measurements from the multi-target scene. Based on the nodes’ exchange measurements and other data, the WSN positioning scenarios can be divided into cooperative and noncooperative. Compared with traditional positioning methods, cooperative (also known as collaborative) positioning has important research and application significance. For instance, cooperative positioning of connected vehicles that effectively utilizes the relative observations from the vehicle-to-vehicle (V2V) devices has become a significant trend of future cooperative intelligent transportation system (ITS) applications [5]. Its advantages have been confirmed theoretically and algorithmically [6,7]. The analysis of Fisher information can show that nodes can obtain better positioning accuracy and availability in cooperative scenarios [8].

For various ground systems, the primary positioning methods can be divided into four categories, among which the distance-based time of arrival (TOA) and time difference of arrival (TDOA) positioning methods are more common [9]. At present, various classic algorithms for cooperative positioning are available, such as maximum likelihood (ML) estimation [10], extended Kalman filter (EKF) [11], particle filter (PF) [12], etc. These algorithms use the minimum mean squared error (MESE) as the evaluation criterion of estimation, and under certain conditions, they are essentially equivalent to the LS estimator. For instance, when the ranging error has a Gaussian distribution, the ML algorithm is equivalent to the LS estimator.

### 1.1. Related Works

When estimating the location of user nodes, it is necessary to select a reasonable initial value. Unfortunately, sometimes, there is a problem with initial value sensitivity when the algorithms are used. In addition, the NLOS propagation error of the signal is also an important factor affecting the positioning accuracy. Thus far, some efforts have been made to solve these problems. By way of observation, if measurements based on the received signal strength (RSS) and angle of arrival (AOA) are considered as the auxiliary positioning methods, the NLOS effect can be suppressed by the algorithms, and the positioning accuracy can be improved [13,14,15,16]. In general, hybrid positioning methods with multiple measurement methods tend to have higher positioning accuracy and be more robust than those based on a single measurement. The localization based on mmWave communication is a hybrid positioning method, but differs from the traditional approach. Its advantage lies in the unique structure of the MIMO systems, so it can determine the position of targets using only one base station by measuring the angles of both the transmitted and received signals [17]. This scheme reduces the cost of base station deployment, but also has apparent disadvantages. One major problem is the diffuse reflection effect of the signal, which causes a higher angle measurement error. For this reason, the positioning results will not be accurate; this topic remains to be studied, and the related improvements are yet to be attained [18,19].

As for the algorithms, a variety of robust positioning algorithms are available. The localization technique based on multidimensional scaling (MDS), which was first proposed by Shang et al. [20], offers a new solution of node localization. Recently, several localization methods related to the classical MDS method have been applied to sensor networks. Forero and Giannakis presented a robust multidimensional scaling based on regularized least squares [21]. Focusing on the problem of localization in mixed LOS and NLOS scenarios, a novel localization algorithm called the Gaussian mixed model based non-metric multidimensional (GMDS) was proposed [22]. The advantages of MDS are that one can obtain actual positions between nodes by setting only a few anchor nodes; besides, the anchor nodes’ deployment has no strict restriction. However, it is unreliable in large-scale networks with sparse connectivity. The work of Destino and Abreu transformed the original WLS function into a convex one by introducing the Gaussian kernel function and optimizing the smoothing parameters [23]. In the literature [24,25], the problem has been formulated by applying robust statistics techniques on squared range measurements. This provides the opportunity to find the estimate efficiently. However, this formulation is not optimal in the ML sense [23]. Another class of methods is based on the convex relaxation technique. The paper [26] derived a maximum likelihood estimator for Laplacian noise and relaxed it to a convex program by linearizing and dropping a rank constraint. Soares et al. [27] set forth a convex underestimator of the maximum likelihood cost for the sensor network localization based on the convex envelopes of its parcels. At the same time they capitalized on the robust estimation properties of the Huber function and derived a convex relaxation [28]. It is known that the semidefinite programming (SDP) algorithm is one of the most used methods, which also transforms the position model into a convex optimization problem by applying the convex relaxation technique [29,30,31]. These approaches can not only limit the errors caused by the NLOS effect, but also make the cost function convex. In other words, they are insensitive to the initial value’s selection. However, the downside of these algorithms is that the estimation accuracy will decrease slightly. Besides, other approaches such as the parallel projection method (PPM) [19,32], projection onto convex sets (POCS) [33,34], etc., have been proposed. These two methods turn the LS cost function into a convex one. At the same time, the PPM can be used in the distributed cooperative scenario, which significantly reduces the pressure of information interaction between the two nodes. An outlier detection method was proposed by Wang et al. [35] based on the maximum entropy principle and fuzzy set theory.

### 1.2. Contributions

It is known that the LS cost function is nonlinear and nonconvex in the global region. We consider the location result of an iterative algorithm depending on the selected initial value if the function has more than one local optimal point, and it seems that the initial value selection is associated with the convexity of the cost function. Existing studies analyzed the LS source localization problem to determine the condition of the function being convex [36,37]. Similarly, for WSN localization, the convexity or the number of local extremum points seems to relate to the quantity of targets and the ranging error. To explore this problem further, we perform a study of the LS model for WSN localization to understand theoretically how the targets and the ranging error affect the extremum point. The major contributions of the paper are as follows:

- A sufficient and unnecessary condition for the LS cost function to be convex is proposed and proven for WSN positioning.
- We define the relative angle matrix for both noncooperative and cooperative scenarios and show that the LS function can be transformed into a globally convex function if all the targets are located inside the convex polygon formed by its adjacent nodes.
- A robust algorithm that detects the relative angular matrix is proposed for WSN localization. Additionally, we improve the algorithm by using angle constraints so that it can be used in both the AOA/TOA and TOA positioning methods, which extends the applicability of the method.

It is worth noting that with the development of MIMO technology, the acquisition of ranging information and angle information between two nodes becomes easier, as the technology provides hardware and technical support for the measurement of the relative angular matrix. On the other hand, the position of the virtual anchors can be calculated, so mmWave positioning can be transformed into an AOA/TOA positioning model in the traditional sense, and the null space algorithm improved in this paper can be used to solve the position.

The rest of the paper is organized as follows. In Section 2, some basic definitions and model descriptions are given. In Section 3, we analyze the convexity of the unconstrained LS positioning model and derive a sufficient and unnecessary condition for the function to be convex. In Section 4, we first provide the definition of the relative angular matrix, then subsequently prove some important properties, and propose a novel null space algorithm by adding the angle constraint. We perform a numerical simulation that aims to verify the correctness of the proposition and evaluate the performance of the proposed algorithm in Section 5. Finally, we conclude the paper in Section 6.

## 2. Definition and Scenario Description

### 2.1. Definition of the Nodes and Links

In the wireless network location scenario based on the TOA method, we assume there are *M* targets and use the set $F_{c}=\left\{T_{1},T_{2},\ldots ,T_{M}\right\}$ to enumerate them. The real coordinates of a target are $x_{j}\in R^{\eta },\;\;1\leq j\leq M,\;\;j\in N^{+}$, where $\eta \in N^{+}$ is the Euclidean spatial dimension of the location scene. There are *N* anchors, which are represented by the set $F_{a}=\left\{A_{1},A_{2},\ldots ,A_{N}\right\}$, and their real coordinates are $s_{i}\in R^{\eta },\;\;1\leq i\leq M,\;\;i\in N^{+}$. Let the set of all node records be $F_{t}$; then, $F_{t}=F_{a}\cup F_{c}$. Assume that there is a total of *L* ranging links in the positioning scenario and the set of links is $L=\left\{l_{1},l_{2},\ldots ,l_{L}\right\}$. In the cooperative scenario, the ranging links can be divided into two categories: that of ranging links between targets and anchors (AT links) and that of cooperative ranging links between two targets (TT links). The set of all distance observations is denoted by $D_{t}$, the set of AT links by $D_{a}$, and the set of TT links by $D_{c}$. Obviously, we have $D_{t}=D_{a}\cup D_{c}$, $D_{a}\cap D_{c}=\emptyset$, $\left|D_{t}\right|=L$, and $\left|D_{a}\right|=N$. Let $d_{K_{i}K_{j}}$ and ${\hat{d}}_{K_{i}K_{j}}$ be the real and estimated distances between the nodes $K_{i}$ and $K_{j}$.

### 2.2. Definition of the Errors

In the TOA positioning method, since the signal is affected by noise, the multipath effect, and the NLOS effect during propagation, the observed value is not the real distance between the two nodes, and there usually exists a ranging error. Let $\varepsilon ={\left[\varepsilon_{1}\;\;\varepsilon_{2}\ldots \;\;\varepsilon_{L}\right]}^{T}\in R^{L}$ be the ranging error vector, where $\varepsilon_{i}$ represents the error on the ith link. In the LOS environment, the ranging error is caused entirely by noise, which usually follows a Gaussian distribution with a mean of zero and a constant variance. Here, we denote it by $\varepsilon_{los}$. In the NLOS environment, besides the noise, there also exists a positive deviation that follows the Gaussian distribution with both the mean and variance being constant. Here, we denote it by $\varepsilon_{nlos}$. Then, the ranging error can be modeled as follows:

(1) $$\varepsilon_{i}=\left\{\begin{array}{cc} \varepsilon_{los}, & \mathrm{if}\;\mathrm{the}\;i\mathrm{th}\;\mathrm{path}\;\mathrm{is}\;\mathrm{LOS}\\ \varepsilon_{los}+\varepsilon_{nlos}, & \mathrm{if}\;\mathrm{the}\;i\mathrm{th}\;\mathrm{path}\;\mathrm{is}\;\mathrm{NLOS} \end{array}\right.$$

where $\varepsilon_{los}∼N\left(0,\sigma_{los}^{2}\right)$, $\varepsilon_{nlos}∼N\left(\mu_{nlos},\sigma_{nlos}^{2}\right)$, and $\mu_{nlos}>0$.

In the AOA method, we assume that there are observation errors in the relative angle matrix Ω, which will be defined in the fourth part of this paper. Hence,

(2) $$\tilde{\Omega }=\Omega +\Delta$$

where $\tilde{\Omega }$ represents the observation of the relative angular matrix and Δ is the error matrix, an element $\delta_{ij}$ of which follows the Gaussian distribution $\delta_{ij}∼N\left(\mu_{\alpha },\sigma_{\alpha }^{2}\right)$.

### 2.3. Noncooperative Scenario Description

In the noncooperative localization scenario, there are *M* targets, and there is no link between any two targets. Consider target $T_{j}$; the target’s real and estimated positions are $x_{j}$ and ${\hat{x}}_{j}$; then, the distance can be given by:

(3) $$\left\{\begin{array}{c} d_{T_{j}A_{i}}={∥s_{i}-x_{j}∥}_{2}\\ {\hat{d}}_{T_{j}A_{i}}={∥s_{i}-{\hat{x}}_{j}∥}_{2} \end{array}\right.$$

where ${∥\cdot ∥}_{2}$ represents the Euclidean distance between two nodes. In the noncooperative scenario, there are *N* ranging links that are denoted by $l_{1},l_{2},\ldots ,l_{N}$, respectively. Let $d_{i}$ and ${\hat{d}}_{i}$ be the real and estimated distances, corresponding to link $l_{i}$. Then, we can assign the values as follows:

(4) $$d_{i}=\left\{\begin{array}{c} d_{T_{1}A_{i}}\;\;\;\;\;\;\;\;\;\;1\leq i\leq q_{1a}\\ d_{T_{j}A_{i}}\;\;\;\;\sum_{m=1}^{j-1}q_{ma}<i\leq \sum_{m=1}^{j}q_{ma}\;\;\;\;\;\;j\geq 2 \end{array}\right.,$$

(5) $${\hat{d}}_{i}=\left\{\begin{array}{c} {\hat{d}}_{T_{1}A_{i}}\;\;\;\;\;\;\;\;\;\;1\leq i\leq q_{1a}\\ {\hat{d}}_{T_{j}A_{i}}\;\;\;\;\sum_{m=1}^{j-1}q_{ma}<i\leq \sum_{m=1}^{j}q_{ma}\;\;\;\;j\geq 2 \end{array}\right.,$$

where $1\leq i\leq N,\;\;i\in N^{+}$ and $q_{ja}$ is the quantity of all links directly connected to node $T_{j}$. Assume that the distance observations are $\rho_{i}\in D_{a},\;1\leq i\leq N,\;\;i\in N^{+}$. If the observation error is considered, the relationship between the distance observation and the true distance is given by:

(6) $$\rho_{i}=d_{i}+\varepsilon_{i}.$$

Accordingly, the unconstrained LS estimation model in the noncooperative scenario can be expressed as follows:

(7) $$arg\;\;\min_{{\hat{d}}_{i}}\;\;F_{s}=\sum_{i=1}^{N}{\left({\hat{d}}_{i}-\rho_{i}\right)}^{2}.$$

### 2.4. Cooperative Scenario Description

The cooperative scenario is very similar to the noncooperative one. The difference is that there exist ranging and information interactions between two targets. Let $x_{j}$ and ${\hat{x}}_{j}$ be the real and estimated coordinates of node $T_{j}$; then,

(8) $$\left\{\begin{array}{c} d_{T_{j}A_{i}}={∥s_{i}-x_{j}∥}_{2}\\ {\hat{d}}_{T_{j}A_{i}}={∥s_{i}-{\hat{x}}_{j}∥}_{2} \end{array}\right.,\;\;\;\;\;\left\{\begin{array}{c} d_{T_{j}T_{k}}={∥x_{k}-x_{j}∥}_{2}\\ {\hat{d}}_{T_{j}T_{k}}={∥{\hat{x}}_{k}-{\hat{x}}_{j}∥}_{2} \end{array}\right.,\;\;\;k>j.$$

In the cooperative scenario, there are *L* ranging links in total, which are denoted by $l_{1},l_{2},\ldots ,l_{L}$. Let $d_{i}$ and ${\hat{d}}_{i}$ be the real and estimated distances corresponding to link $l_{i}$. Next, we can assign the values as follows. If $1\leq i\leq N,i\in N^{+}$, each $l_{i}$ is an AT link, and we assign the values according to Formulas (4) and (5). If $N<i\leq L,i\in N^{+}$, $l_{i}$ is a TT link, and we assign the values as follows:

(9) $$d_{i}=\left\{\begin{array}{c} d_{T_{1}T_{k}},\;\;\;\;\;\;\;\;\;\;N+1\leq i\leq q_{1c}\\ d_{T_{j}T_{k}},\;\;N+\sum_{m=1}^{j-1}q_{mc}<i\leq N+\sum_{m=1}^{j}q_{mc}\;\;\;\;j\geq 2 \end{array}\right.,$$

(10) $${\hat{d}}_{i}=\left\{\begin{array}{c} {\hat{d}}_{T_{1}T_{k}},\;\;\;\;\;\;\;\;\;\;N+1\leq i\leq q_{1c}\\ {\hat{d}}_{T_{j}T_{k}},\;\;N+\sum_{m=1}^{j-1}q_{mc}<i\leq N+\sum_{m=1}^{j}q_{mc}\;\;\;\;j\geq 2 \end{array}\right..$$

Above, $q_{jc}$ is the quantity of TT links with endpoints $T_{j},T_{k}$ such that k>j. Additionally, there also are $T_{k}\in U_{j}$, and $U_{j}$ is the set of targets that have ranging links with $T_{j}$. In set $U_{j}$, the elements’ subscripts are arranged in increasing order, and $T_{k}$ is the gth element, the index of which is calculated by:

(11) $$g=\left\{\begin{array}{cc} i-N, & j=1\\ i-N-\sum_{m=1}^{j-1}q_{mc}, & j\geq 2 \end{array}\right..$$

Let $\rho_{i}\in D_{t},\;1\leq i\leq L,\;\;i\in N^{+}$ be distance observations; then, the relationship between $\rho_{i}$ and $d_{i}$ satisfies Formula (6). Accordingly, in the cooperative scenario, the unconstrained LS model is constructed as:

(12) $$arg\;\;\min_{{\hat{d}}_{i}}\;\;F_{c}=\sum_{i=1}^{L}{\left({\hat{d}}_{i}-\rho_{i}\right)}^{2}.$$

In this paper, we refer to $F_{s}$ and $F_{c}$ together as the LS positioning cost function and denote it by *F*.

## 3. Convex Analysis of the Model

In the previous part, we presented the unconstrained LS localization model in the cooperative and noncooperative scenarios. For the positioning problem, we are interested in finding the global optimum of the cost function. Due to the nonconvex property of the LS function, there may be multiple local optima or stagnation points in most scenes. As a result, a local minimum solution is obtained in the iterative process, which affects the positioning accuracy significantly. In this part, we discuss the nonconvex property of the unconstrained LS localization model to study when the cost function is convex and when it is nonconvex. To simplify the analysis, it may be worth considering the positioning problem on the two-dimensional plane, i.e., for η=2. It is convenient to generalize the conclusion to a higher dimensional space. Let $s_{j}={\left[x_{s_{i}}\;y_{s_{i}}\right]}^{T}$ be the coordinates of the anchor and $x_{j}={\left[x_{j}\;y_{j}\right]}^{T}$ be the coordinates of the target. At the same time, the corresponding estimated position is ${\hat{x}}_{j}={\left[{\hat{x}}_{j}\;{\hat{y}}_{j}\right]}^{T}$.

### 3.1. Analysis of the Noncooperative Scenario

It is known that the convex property of a function is directly related to its Hessian matrix, and the following theorem holds:

**Theorem** **1.** *The second-order condition ensures the function is convex. Assume that function f of x is second-order differentiable, and let its domain be domf. If domf is a convex set and the Hessian matrix exists, then a sufficient and necessary condition for the function to be convex in domf is that, for ∀x∈domf, its Hessian matrix is a semipositive definite matrix [38].*

To simplify the problem, we first consider the scenario of only one target, and the target number is $T_{1}$. In this case, there are *N* links between $T_{1}$ and the anchors. We try to compute the gradient (the first-order differential) and the Hessian matrix (the second-order differential) of the cost function $F_{s}$. The calculation results are shown in Equations (13) and (14).

(13) $$grad\;F_{s}=\nabla_{{\hat{x}}_{1}}=2\sum_{i=1}^{N}\left[\begin{array}{c} \frac{\left({\hat{d}}_{i}-\rho_{i}\right)\left(x_{s_{i}}-{\hat{x}}_{1}\right)}{{\hat{d}}_{i}}\\ \frac{\left({\hat{d}}_{i}-\rho_{i}\right)\left(y_{s_{i}}-{\hat{y}}_{1}\right)}{{\hat{d}}_{i}} \end{array}\right],$$

(14) $$\nabla_{{\hat{x}}_{1}}^{2}=2\sum_{i=1}^{N}\left[\begin{array}{cc} \frac{{\hat{d}}_{i}-\rho_{i}}{{\hat{d}}_{i}}+\frac{\rho_{i}}{{\hat{d}}_{i}^{3}}{\left(x_{s_{i}}-{\hat{x}}_{1}\right)}^{2} & \frac{\rho_{i}}{{\hat{d}}_{i}^{3}}\left(x_{s_{i}}-{\hat{x}}_{1}\right)\left(y_{s_{i}}-{\hat{y}}_{1}\right)\\ \frac{\rho_{i}}{{\hat{d}}_{i}^{3}}\left(x_{s_{i}}-{\hat{x}}_{1}\right)\left(y_{s_{i}}-{\hat{y}}_{1}\right) & \frac{{\hat{d}}_{i}-\rho_{i}}{{\hat{d}}_{i}}+\frac{\rho_{i}}{{\hat{d}}_{i}^{3}}{\left(y_{s_{i}}-{\hat{y}}_{1}\right)}^{2} \end{array}\right].$$

Considering Theorem 1, we obtain the following corollary:

**Corollary** **1.** *If $dom\;\;F_{s}$ of the LS cost function is $R^{2}$, it obviously is a convex set. Assume that the set of all ${\hat{x}}_{1}$, which satisfy condition $\nabla_{{\hat{x}}_{1}}^{2}⪰0$, is A. Then, for ${\hat{x}}_{1}\in A$, $F_{s}$ is a convex function.*

Corollary 1 is a sufficient and necessary condition for $F_{s}$ to be convex. Finding a set A that satisfies the condition is equivalent to dividing the domain in $R^{2}$ so that $F_{s}$ is convex in each divided subinterval. Next, the condition of $\nabla_{{\hat{x}}_{1}}^{2}⪰0$ will be further analyzed. Note that $\nabla_{{\hat{x}}_{1}}^{2}$ is a real symmetric matrix, and the two following lemmas hold:

**Lemma** **1.** *The eigenvalues of a real symmetric matrix are all real numbers.*

**Lemma** **2.** *A real symmetric matrix is a semipositive definite matrix if and only if all its eigenvalues are nonnegative [39].*

Lemma 2 shows that the assessment of whether a matrix is positive semidefinite can be transformed into the determination of whether the eigenvalues are positive or negative. Hence, we consider calculating the eigenvalues of the Hessian matrix. Let $J=\nabla_{{\hat{x}}_{1}}^{2}\in R^{2\times 2}$, and let λ be the eigenvalue of J; then, the eigenvalue polynomial of J is:

(15) $$G=\left|\begin{array}{cc} \lambda -J_{11} & -J_{12}\\ -J_{21} & \lambda -J_{22} \end{array}\right|,$$

where $J_{ij}$ are the elements of J. Let G=0; then, the following characteristic polynomial equation can be obtained:

(16) $$\lambda^{2}-\left(J_{11}+J_{22}\right)\lambda +J_{11}J_{22}-J_{12}J_{21}=0.$$

It is a quadratic equation of one variable. From Lemma 1, we observe that there must be two real roots, and the discriminant of roots Δ≥0 is invariable. From the distribution relation of the two roots, the sufficient and necessary condition of having two nonnegative real roots is:

(17) $$\left\{\begin{array}{c} J_{11}+J_{22}>0\\ J_{11}J_{22}-J_{12}J_{21}\geq 0 \end{array}\right..$$

Therefore, we can derive the following corollary:

**Corollary** **2.** *Set A consists of all the sets of ${\hat{x}}_{1}$ that satisfy the condition of the inequality group (15).*

Corollary 2 is an equivalent condition of the LS cost function $F_{s}$ being convex. Compared with Corollary 1, Corollary 2 transforms the condition that the Hessian matrix is semipositive definite into the solution of the inequality system, which provides a feasible method for finding the set satisfying the requirements. However, due to the nonlinear characteristics of Equation (17), it is difficult to obtain the analytic solutions of inequalities. In the next part, we discuss a particular case and try to provide the proof.

**Proposition** **1.** *If there exists a set C in which all the estimates ${\hat{x}}_{1}$ satisfy ${\hat{d}}_{i}-\rho_{i}\geq 0$, then the LS cost function $F_{s}$ is convex and C⊆A.*

The proof of Proposition 1 is given in Appendix A.

**Figure 1:**
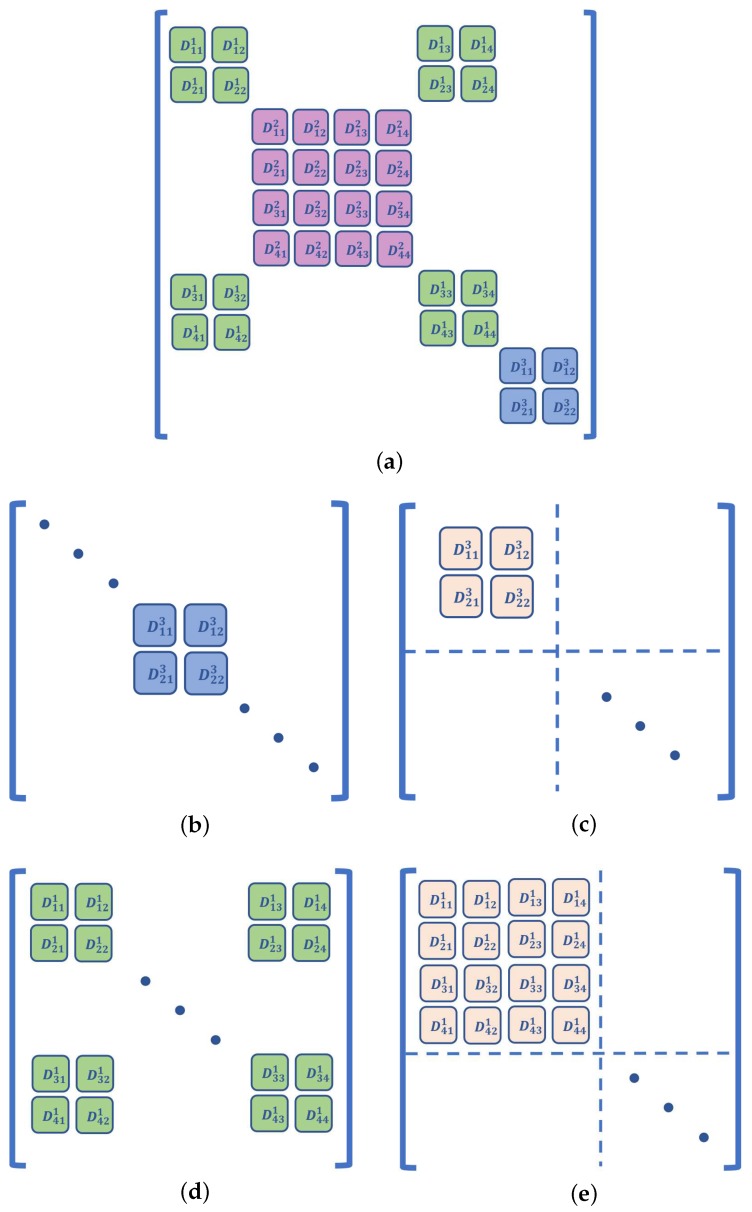
A simple example of Hessian matrix J. Graph (**a**) shows the arrangement of elements in one of the cooperative scenarios. Graph (**b**) represents $Q_{i}$ associated with the target $T_{i}$, and (**c**) represents $Q_{i}^{\prime }$, which is the matrix after the row and column transformation of $Q_{i}$. Similarly, Graphs (**d**,**e**) represent $Q_{i}$ and $Q_{i}^{\prime }$ associated with target-target (TT) links in the cooperative scenario.

If there is more than one target and their number is *M*, the Hessian matrix J has 2M rows and 2M columns, i.e., $J\in R^{2M\times 2M}$. Consider a decomposition of J, and let $J=\sum_{i=1}^{N}Q_{i}$. In the preceding formula, $Q_{i}$ is the Hessian matrix corresponding to the ranging link $l_{i}$. It is easy to observe that there are four elements, and the elements of $Q_{i}$ are located on the diagonal or adjacent positions of J. An example is shown in Figure 1b, where $D_{ij}^{k}$ represents the corresponding elements in J, which are the second-order partial derivatives of $F_{s}$ with respect to the coordinates of a target. We translate the four elements in $Q_{i}$ to the upper left corner via the elementary row-column transformation of matrices. Let the transformed matrix be $Q_{i}^{\prime }$, as shown in Figure 1c; we observe that $Q_{i}$ and $Q_{i}^{\prime }$ are similar matrices. The following lemma holds for similar matrices:

**Lemma** **3.** *Similar matrices have the same eigenvalues.*

This shows that $Q_{i}$ and $Q_{i}^{\prime }$ have the same eigenvalues. Let $G_{i}^{\prime }=\lambda I-Q_{i}^{\prime }$; to obtain the eigenvalues of $G_{i}^{\prime }$, we construct the following block for $G_{i}^{\prime }$:

(18) $$G_{i}^{\prime }=\left[\begin{array}{cc} G_{11} & G_{12}\\ G_{21} & G_{22} \end{array}\right].$$

Above, $G_{11}=\left[\begin{array}{cc} \lambda -g_{11} & -g_{12}\\ -g_{21} & \lambda -g_{22} \end{array}\right]\in R^{2\times 2}$, $G_{12}=0\in R^{2\times \left(2M-2\right)}$, $G_{21}=0\in R^{\left(2M-2\right)\times 2}$, $G_{22}=\Lambda \in R^{\left(2M-2\right)\times \left(2M-2\right)}$, where $g_{kl}$ is the element at the kth row and lth column of $G_{i}^{\prime }$. From the determinant theorem of block matrices, we know that:

(19) $$|G_{i}^{\prime }\left|=\right|G_{11}\left|\cdot \right|G_{22}-G_{21}G_{11}^{-1}G_{12}\left|=\right|G_{11}\left|\cdot \right|G_{22}|=\lambda^{2M-2}\left|G_{11}\right|.$$

Let $|G_{i}^{\prime }|=0$. According to the previous analysis, the two solutions of $|G_{11}|=0$ are λ=1 and $\lambda =\frac{{\hat{d}}_{i}-\rho_{i}}{{\hat{d}}_{i}}$, and the remaining 2M−2 solutions of $|G_{i}^{\prime }|=0$ are all zero. If ${\hat{d}}_{i}-\rho_{i}\geq 0$, then $Q_{i}⪰0$.

Proposition 1 still holds if there is more than one target, and it is a sufficient and unnecessary condition for $F_{s}$ to be locally convex. The nonconvex interval of $F_{s}$ decreases with the increasing $\varepsilon_{i}$ and the number of links that satisfy $\varepsilon_{i}>0$.

### 3.2. Analysis of the Cooperative Scenario

Similarly, we try to find a set of ${\hat{x}}_{j}$ for the condition that the LS cost function is convex in the cooperative scenario. We guess that Proposition 1 is also valid in that scenario and try to prove it. In this scenario, the ranging link consists of two parts, an AT link and a TT link. Inspired by Lemma 3, we decompose Hessian matrix J into several submatrices $Q_{i}$. Each $Q_{i}$ is actually a function related to ${\hat{d}}_{i}$, which is denoted by $f\left({\hat{d}}_{i}\right)={\left({\hat{d}}_{i}-\rho_{i}\right)}^{2}$. The element of $Q_{i}$ is the second-order partial derivative of function $f\left({\hat{d}}_{i}\right)$ with respect to the target coordinates ${\hat{x}}_{j}$. In Section 3.1, it has been proven that Proposition 1 is satisfied if $l_{i}$ is an AT link. Next, we will show that Proposition 1 is also satisfied for TT links. To facilitate the analysis, we select one TT link $l_{k}$ in the cooperative scenario and denote the targets at the end of the link as $T_{m}$ and $T_{n}$, the estimates of which are $\left({\hat{x}}_{m},{\hat{y}}_{m}\right)$ and $\left({\hat{x}}_{n},{\hat{y}}_{n}\right)$, respectively. If the distance measurement is $\rho_{k}$, then the LS cost function can be written as:

(20) $$F_{c}={\left({\hat{d}}_{k}-\rho_{k}\right)}^{2}.$$

Computing the partial derivatives of $F_{c}$ with respect to ${\hat{x}}_{m},{\hat{x}}_{n},{\hat{y}}_{m},{\hat{y}}_{n}$, the gradient vector (the first-order differential) can be obtained as follows:

(21) $$grad\;F_{c}=\nabla_{{\hat{x}}_{m},{\hat{x}}_{n}}=\frac{2\left({\hat{d}}_{k}-\rho_{k}\right)}{{\hat{d}}_{k}}\left[\begin{array}{c} {\hat{x}}_{m}-{\hat{x}}_{n}\\ {\hat{x}}_{n}-{\hat{x}}_{m}\\ {\hat{y}}_{m}-{\hat{y}}_{n}\\ {\hat{y}}_{n}-{\hat{y}}_{m} \end{array}\right].$$

Hence, the Hessian matrix is:

(22) $$J=\nabla_{{\hat{x}}_{m},{\hat{x}}_{n}}^{2}.$$

We are interested in the eigenvalue of J; the characteristic polynomial can be calculated as follows:

(23) $$\lambda^{2}\left(\lambda -4\right)\left(\lambda -4\frac{{\hat{d}}_{k}-\rho_{k}}{{\hat{d}}_{k}}\right)=0.$$

Then, the four eigenvalues can be solved as follows:

(24) $$\lambda_{1}=\lambda_{2}=0,\lambda_{3}=4,\lambda_{4}=4\frac{{\hat{d}}_{k}-\rho_{k}}{{\hat{d}}_{k}}.$$

If ${\hat{d}}_{k}-\rho_{k}\geq 0$, thus a TT link has similar properties to those of an AT link. In the following, we try to generalize this further. If there are two kinds of ranging links in the LS cost function, this property remains unchanged.

Consider the case of *M* targets in the cooperative scenario; the Hessian matrix of $F_{c}$ is $J\in R^{2M\times 2M}$. For example, the elements of the matrix are shown in Figure 1a, where $D_{ij}^{k}$ represents the corresponding element that is the second-order partial derivative of $F_{c}$ with respect to the coordinates of a target. Consider a decomposition of J, and let $J=\sum_{i=1}^{L}Q_{i}$. In the preceding formula, $Q_{i}$ is the Hessian matrix corresponding to the ranging link $l_{i}$. If $l_{i}$ is an AT link, the analysis and result shown in Section 3.1 apply. If the ranging link is a TT link, there are sixteen elements in each $Q_{i}$. An example is shown in Figure 1d. All the elements in $Q_{i}$ are translated to the upper left corner, as shown in Figure 1e. We denote the transformed matrix by $Q_{i}^{\prime }$ and let $G_{i}^{\prime }=\lambda I-Q_{i}^{\prime }$. Similarly, we construct the following block for $G_{i}^{\prime }$:

(25) $$G_{i}^{\prime }=\left[\begin{array}{cc} G_{11} & G_{12}\\ G_{21} & G_{22} \end{array}\right].$$

Above, $G_{11}=\left[\begin{array}{ccc} \lambda -g_{11} & \ldots & -g_{14}\\ \vdots & \ddots & \vdots \\ -g_{41} & \ldots & \lambda -g_{44} \end{array}\right]\in R^{4\times 4}$, $G_{12}=0\in R^{4\times \left(2M-4\right)}$, $G_{21}=0\in R^{\left(2M-4\right)\times 4}$, and $G_{22}=\Lambda \in R^{\left(2M-4\right)\times \left(2M-4\right)}$, and $g_{kl}$ is the element at the *k*th row and the *l*th column of $G_{i}^{\prime }$. Similar to Formula (23), we have:

(26) $$|G_{i}^{\prime }\left|=\right|G_{11}\left|\cdot \right|G_{22}-G_{21}G_{11}^{-1}G_{12}\left|=\right|G_{11}\left|\cdot \right|G_{22}|=\lambda^{2M-4}\left|G_{11}\right|.$$

Let $|G_{i}^{\prime }|=0$. Reviewing the analysis for TT links, the four solutions of $|G_{11}|=0$ are $\lambda_{1}=\lambda_{2}=0$, $\lambda_{3}=4$, and $\lambda_{4}=4\frac{{\hat{d}}_{i}-\rho_{i}}{{\hat{d}}_{i}}$, and the remaining 2M−4 solutions of $|G_{i}^{\prime }|=0$ are all zero. Hence, if ${\hat{d}}_{i}-\rho_{i}\geq 0$, then $Q_{i}⪰0$.

The analysis above shows that Proposition 1 is still valid in the cooperative scenario. Similar to the noncooperative scenario, the nonconvex interval of $F_{c}$ decreases with the increasing $\varepsilon_{i}$ and the number of links that satisfy $\varepsilon_{i}>0$.

Proposition 1 shows that if the condition ${\hat{d}}_{i}-\rho_{i}\geq 0$ is satisfied, an appropriate interval in which the LS cost function *F* is convex can always be found. Based on this, we can describe the condition for *F* to be convex globally.

**Proposition** **2.** *For ∀i, if ${\hat{d}}_{i}-\rho_{i}\geq 0$ always holds on $\hat{x}={\left[{\hat{x}}_{1}\;{\hat{x}}_{2}\;\ldots \;{\hat{x}}_{M}\right]}^{T}\in R^{2M}$, then F is convex on $R^{2M}$.*

Proposition 2 is a sufficient and unnecessary condition for the global convexity of the LS cost function. In a practical scenario of the ranging error being greater than zero, the distance observation $\rho_{i}$ is positive. In this case, the condition of global convexity is not satisfied. If $\varepsilon_{i}\leq -d_{i}$, we have $\rho_{i}\leq 0$; then, ${\hat{d}}_{i}-\rho_{i}\geq 0$ is invariable, and *F* is convex in the global range. Although the global convexity condition is satisfied if all $\varepsilon_{i}\leq -d_{i}$, it is a low probability event that all the observation values will be negative because of the independence between the distance measurement errors. Therefore, in an actual location determination scenario, it is not a common phenomenon for the LS cost function to be convex in the global range.

## 4. Null Space of the Relative Angle Matrix

From the analysis in Section 3, it is known that a positive ranging error will cause the LS cost function to be nonconvex. In this condition, when the iterative method is used to search for the optimal solution, it may fall into a local minimum, causing the result obtained to not be the optimal global solution. There are generally two ways of solving this problem. The first is to divide the domain $R^{2M}$ into several intervals so that the cost function is convex in each subinterval. Then, the appropriate initial value is selected in each subinterval, and the result is obtained by the iterative method. Proposition 1 shows that such subintervals must exist, so this method is feasible in any case. The second method is to modify the original LS localization model to make it convex in the global range. The advantage of this method is that the convexity weakens the requirement of initial value selection. That is, the solution is insensitive to initial value selection. Based on Proposition 2, we propose a robust method using the relative angle matrix for WSN positioning. The basic idea of this method is to transform the LS cost function into a globally convex function by calculating the relative angle matrix. This algorithm ensures that the minimum obtained by the Gauss–Newton iteration method will be the optimal global solution. Some further analysis will also be performed in this part.

### 4.1. Definition of the Relative Angle Matrix

**Definition** **1.** *The relative angle matrix in the noncooperative scenario is defined as:*

(27) $$\Omega_{s}\overset{△}{=}\left[\begin{array}{cccc} 1 & \cos\left(\theta_{1T2}\right) & \ldots & \cos\left(\theta_{1TN}\right)\\ \cos\left(\theta_{2T1}\right) & 1 & \ddots & \cos\left(\theta_{2TN}\right)\\ \vdots & \ddots & \ldots & \vdots \\ \cos\left(\theta_{1TN}\right) & \cos\left(\theta_{2TN}\right) & \ldots & 1 \end{array}\right],$$

*where $\Omega_{s}\in R^{N\times N}$. If $\theta_{iTj}^{\prime }$ is the angle between $l_{i}$ and $l_{j}$, then $\theta_{iTj}$ is given by:*

(28) $$\theta_{iTj}=\left\{\begin{array}{c} \theta_{iTj}^{\prime }\;,\;l_{i}\cap l_{j}=T_{k}\\ \frac{\pi }{2}\;,\;\;l_{i}\cap l_{j}\neq T_{k} \end{array}\right..$$

*Assume the following formulation:*

(29) $$P_{s}=\left[\begin{array}{ccccc} \frac{x_{s_{1}}-{\hat{x}}_{1}}{{\hat{d}}_{1}} & \frac{y_{s_{1}}-{\hat{y}}_{1}}{{\hat{d}}_{1}} & \ldots & 0 & 0\\ \frac{x_{s_{2}}-{\hat{x}}_{1}}{{\hat{d}}_{2}} & \frac{y_{s_{2}}-{\hat{y}}_{1}}{{\hat{d}}_{2}} & \ldots & 0 & 0\\ \vdots & \vdots & \ddots & \vdots & \vdots \\ 0 & 0 & \ldots & \frac{x_{s_{N}}-{\hat{x}}_{M}}{{\hat{d}}_{N}} & \frac{y_{s_{N}}-{\hat{y}}_{M}}{{\hat{d}}_{N}} \end{array}\right],\;\varepsilon_{s}=\left[\begin{array}{c} \rho_{1}-{\hat{d}}_{1}\\ \rho_{2}-{\hat{d}}_{2}\\ \;\;\;\;\;\;\;\vdots \\ \rho_{N}-{\hat{d}}_{N} \end{array}\right],$$

*where $P_{s}\in R^{N\times 2M}$ and $\varepsilon_{s}\in R^{N}$. Let $\nabla_{{\hat{x}}_{1}}=0$; this condition is equivalent to:*

(30) $$P^{T}\varepsilon_{s}=0.$$

*Further, the relationship between relative angle $\Omega_{s}$ and $P_{s}$ is as follows:*

(31) $$\Omega_{s}=P_{s}P_{s}^{T}.$$

*Substituting (30) into (31), we obtain:*

(32) $$\Omega_{s}\varepsilon_{s}=0.$$

Imitating the noncooperative scenario, we can define the relative angle matrix in the cooperative scenario.

**Definition** **2.** *The relative angle matrix in the cooperative scenario is defined as:*

(33) $$\Omega_{c}\overset{△}{=}\left[\begin{array}{ccccc} 1 & \cos\theta_{1T2} & \cos\theta_{1T3} & \ldots & \cos\theta_{1TL}\\ \cos\theta_{2T1} & 1 & \cos\theta_{2T3} & \ldots & \cos\theta_{2TL}\\ \vdots & \vdots & \ddots & \vdots & \vdots \\ \cos\theta_{L-1T1} & \cos\theta_{L-1T2} & \cos\theta_{L-1T3} & \ldots & \cos\theta_{L-1TL}\\ \cos\theta_{LT1} & \cos\theta_{L-2} & \cos\theta_{LT3} & \ldots & 2 \end{array}\right],$$

*where $\Omega_{c}\in R^{L\times L}$; when i≠j, if $\theta_{iTj}^{\prime }$ is the angle between $l_{i}$ and $l_{j}$, then $\theta_{iTj}$ is defined by Formula (28). If i=j, let $\omega_{ij}$ represent the element of the ith row and jth column in $\Omega_{c}$. If $l_{i}$ is an AT link, then $\omega_{ij}=1$; otherwise, $l_{i}$ is a TT link, and then, $\omega_{ij}=2$. Assume that:*

(34) $$P_{c}=\left[\begin{array}{ccccccc} \frac{x_{s_{1}}-{\hat{x}}_{1}}{{\hat{d}}_{1}} & \frac{y_{s_{1}}-{\hat{y}}_{1}}{{\hat{d}}_{1}} & 0 & 0 & \ldots & 0 & 0\\ \frac{x_{s_{2}}-{\hat{x}}_{1}}{{\hat{d}}_{2}} & \frac{y_{s_{2}}-{\hat{y}}_{1}}{{\hat{d}}_{2}} & 0 & 0 & \ldots & 0 & 0\\ \vdots & \vdots & \vdots & \vdots & \ddots & \vdots & \vdots \\ 0 & 0 & 0 & 0 & \ldots & \frac{x_{s_{N-1}}-{\hat{x}}_{M}}{{\hat{d}}_{N-1}} & \frac{y_{s_{N-1}}-{\hat{y}}_{M}}{{\hat{d}}_{N-1}}\\ 0 & 0 & 0 & 0 & \ldots & \frac{x_{s_{N}}-{\hat{x}}_{M}}{{\hat{d}}_{N}} & \frac{y_{s_{N}}-{\hat{y}}_{M}}{{\hat{d}}_{N}}\\ \frac{{\hat{x}}_{2}-{\hat{x}}_{1}}{{\hat{d}}_{N+1}} & \frac{{\hat{y}}_{2}-{\hat{y}}_{1}}{{\hat{d}}_{N+1}} & \frac{{\hat{x}}_{1}-{\hat{x}}_{2}}{{\hat{d}}_{N+1}} & \frac{{\hat{y}}_{1}-{\hat{y}}_{2}}{{\hat{d}}_{N+1}} & \ldots & 0 & 0\\ \vdots & \vdots & \vdots & \vdots & \ddots & \vdots & \vdots \\ 0 & 0 & \frac{{\hat{x}}_{k}-{\hat{x}}_{2}}{{\hat{d}}_{L}} & \frac{{\hat{y}}_{k}-{\hat{y}}_{2}}{{\hat{d}}_{L}} & \ldots & 0 & 0 \end{array}\right],\;\varepsilon_{c}=\left[\begin{array}{c} \rho_{1}-{\hat{d}}_{1}\\ \rho_{2}-{\hat{d}}_{2}\\ \;\;\vdots \\ \rho_{L}-{\hat{d}}_{L} \end{array}\right],$$

*where $P_{c}\in R^{L\times 2M}$ and $\varepsilon_{c}\in R^{L}$. Then, $\nabla_{{\hat{x}}_{j}}=0$ is equivalent to:*

(35) $$P_{c}^{T}\varepsilon_{c}=0.$$

*Multiplying both ends of the equation by $P_{c}$ results in:*

(36) $$P_{c}P_{c}^{T}\varepsilon_{c}=0.$$

*In the cooperative scenario, it still holds that:*

(37) $$\Omega_{c}=P_{c}P_{c}^{T}.$$

*Substituting Formula (36) into Formula (37), we obtain:*

(38) $$\Omega_{c}\varepsilon_{c}=0.$$

*We refer to the relative angle matrices $\Omega_{s}$ and $\Omega_{c}$ collectively as Ω. The following property of Ω is established generally.*

**Property** **1.** *In the two-dimensional plane, let r be the rank of Ω, i.e., $r=rank\left(\Omega \right)$. Assume that there are M targets in the localization scenario, and the number of unknown variables is τ=2M; then, the inequality r≤τ is satisfied.*

The proof of Property 1 is given in Appendix B.

### 4.2. Null Space Algorithms

From Formulas (32) and (38), we can conclude that the ranging error is the null space of Ω. It is also known from Property 1 that once Ω has been determined, if the number of nontrivial solutions of ranging error ε is $N_{B}$, we have $N_{B}\geq 1$. If and only if r=τ, then $N_{B}=1$. If r<τ, the solution satisfying the equation should be a set, i.e., there is an infinite number of ε satisfying the equation. Hence, if a basic solution of ε has been obtained, ε is the linear space formed by Φ, where $\Phi \in R^{N_{A}\times \left(\tau -r\right)}$. Let $\Phi =\left[\varphi_{1}\;\varphi_{2}\ldots \varphi_{\tau -r}\right]$; then, the general solution of ε can be expressed as:

(39) $$\varepsilon =\sum_{i=1}^{\tau -r}k_{i}\varphi_{i},$$

where $k_{i}\in R$ and $\varphi_{i}\in R^{N_{A}}$. Equation (39) indicates that for ε in the same linear space, the LS cost function *F* has the same local optimal point. However, convexity will vary with ε. If ε>0, *F* is likely to be a nonconvex function; thus, multiple local optima will exist. If ε≪0, it is known from Proposition 2 that *F* can be transformed into a convex function in the global range. Assuming that Ω is known, we can determine Φ for a given ε. If there exists a linear combination of column vectors in Φ that results in ν≪0, then by the same property of the local optima, we can change the LS cost function *F* and make it convex in the global range.

**Proposition** **3.** *Let ν be an element of the null space of* Ω *such that ν≪0; then, Equations (7) and (12) can be rewritten as:*

(40) $$arg\;\;\min_{{\hat{d}}_{i}}\;\;F_{s}^{\prime }=\sum_{i=1}^{N}{\left({\hat{d}}_{i}-\rho_{i}-\nu_{i}\right)}^{2},$$

(41) $$arg\;\;\min_{{\hat{d}}_{i}}\;\;F_{c}^{\prime }=\sum_{i=1}^{L}{\left({\hat{d}}_{i}-\rho_{i}-\nu_{i}\right)}^{2}.$$

In this paper, we refer to $F_{s}^{\prime }$ and $F_{c}^{\prime }$ collectively as $F^{\prime }$. In Proposition 3, $F^{\prime }$ and *F* have the same local optimal point, i.e., the objective functions are equivalent. Additionally, $F^{\prime }$ is also globally convex, which endows it with the characteristic of large-scale convergence. Thus, using it can reduce the sensitivity to the initial value selection. If we want to apply Proposition 3, the following problems remain to be solved:

- How do we achieve Ω?
- For an arbitrary Ω, does ν satisfy the condition of ν≪0?
- When there are errors in Ω, how do we deal with them?

For the first problem, the direct method is to measure the angle, which is similar to the AOA method. In this way, Proposition 3 is transformed into an AOA/TOA hybrid location algorithm. Another method is to transform the distance data into the corresponding angle via the cosine theorem and construct the relative angle matrix. To answer the second question, we will prove that the following properties are valid:

**Property** **2.** *In both noncooperative and cooperative scenarios, there always exists ν≪0 if the node to be located is inside the convex polygon composed of adjacent nodes, whereas there is no ν satisfying this condition if the node is outside the convex polygon.*

The proof of Property 2 is given in Appendix C.

According to Property 2, Proposition 3 can be applied in both noncooperative and cooperative scenarios; however, there is a limitation that it can only be applied if all the targets are located in the convex hull composed of adjacent nodes. In contrast, Proposition 3 does not hold if there are targets outside the convex hull formed by neighboring nodes.

In the third problem, the angle measurement errors will influence the final positioning result. In addition, in the process of calculating the null space of Ω, it is possible that no suitable ν≪0 exists due to the errors. In this case, Proposition 3 will also be invalid, and it is necessary to eliminate it.

The paper [36] used the method of principal component analysis (PCA) to reduce the deviation of the relative angular matrix in source localization. The main steps are as follows. First, Ω is decomposed by SVD. Then, all the eigenvalues are sorted in descending order, and the large eigenvalues are selected as the main eigenvalues. At the same time, the eigenvectors corresponding to the eigenvalues are selected to reconstruct Ω, which is denoted by $\Omega^{\prime }$. The null space of $\Omega^{\prime }$ is determined; one of the vectors is selected as the value of the base vector $\varphi_{1}$ and is multiplied by the coefficient $k_{1}$ to satisfy $\nu =k_{1}\varphi_{1}\ll 0$. The PCA method is simple and efficient; when the measurement errors are not large, it can calculate ν well. In WSN localization, the PCA method cannot obtain the appropriate ν as the errors and the number of targets increase. If the cosine theorem is used to transform the ranging data into angle data, the ranging measurement error will be converted into the angle measurement error after the calculation. Hence, similar problems will also exist. We consider the main reason for this problem to be that the distance circles formed by the node and the range measurement may not intersect at one point. Then, the sum of radian measures of the relative angles of each node at the same point will not equal 2π.

Let ${\hat{\alpha }}_{1},{\hat{\alpha }}_{2},\ldots ,{\hat{\alpha }}_{n}$ be the estimated values of the angles, formed by the target and its adjacent nodes, and the corresponding angle measurements or calculated values be $\alpha_{1},\alpha_{2},\ldots ,\alpha_{n}$. We define the angle least squares cost function as follows:

(42) $$\begin{array}{c} arg\;\;\min_{{\hat{\alpha }}_{i}}\;F_{\alpha }=\sum_{i=1}^{N}{\left({\hat{\alpha }}_{i}-\alpha_{i}\right)}^{2}\\ s.t.\;\sum_{i=1}^{N}{\hat{\alpha }}_{i}=2\pi \end{array}.$$

Formula (42) is a linear optimization problem with equality constraints and can be transformed into an unconstrained optimization problem by introducing Lagrange multipliers. If the Lagrange multiplier is λ, then Formula (42) is equivalent to:

(43) $$arg\;\;\min_{{\hat{\alpha }}_{i}}\;f_{\alpha }^{\prime }=\sum_{i=1}^{N}{\left({\hat{\alpha }}_{i}-\alpha \right)}^{2}+\lambda \left(\sum_{i=1}^{N}{\hat{\alpha }}_{i}-2\pi \right)$$

We calculate the partial derivatives of $f_{\alpha }^{\prime }$:

(44) $$\left\{\begin{array}{c} \frac{\partial f_{\alpha }^{\prime }}{\partial {\hat{\alpha }}_{1}}=2\left({\hat{\alpha }}_{1}-\alpha_{1}\right)-\lambda \\ \;\;\;\;\;\;\;\;\;\;\;\;\vdots \\ \frac{\partial f_{\alpha }^{\prime }}{\partial {\hat{\alpha }}_{n}}=2\left({\hat{\alpha }}_{n}-\alpha_{n}\right)-\lambda \\ \frac{\partial f_{\alpha }^{\prime }}{\partial \lambda }={\hat{\alpha }}_{1}+{\hat{\alpha }}_{2}+\ldots {\hat{\alpha }}_{n}-2\pi \end{array}\right..$$

Setting every derivative in (44) to zero, we obtain the following optimal point:

(45) $$\left\{\begin{array}{c} {\hat{\alpha }}_{1}=\frac{1}{2}\lambda +\alpha_{1}\\ \;\;\;\;\;\;\;\;\;\;\vdots \\ {\hat{\alpha }}_{n}=\frac{1}{2}\lambda +\alpha_{n}\\ \lambda =\frac{2}{n}\left[2\pi -\left({\hat{\alpha }}_{1}+{\hat{\alpha }}_{2}+\ldots {\hat{\alpha }}_{n}\right)\right] \end{array}\right..$$

Using this method, the relative angle between targets and adjacent nodes can be estimated. After that, the sum of relative angular radians of each target will be 2π. According to the properties of the relative angular matrix, ν≪0 must exist. After that, *F* can be transformed into a globally convex function by Proposition 3 and solved by the Gauss–Newton iteration method.

If the cosine theorems are used to calculate the angle, the theorem may be inapplicable. In other words, because of the existence of the ranging error, the triangle’s trilateral side lengths may not satisfy the theorem’s condition, and the method needs to be further improved. In this paper, we use the generalized cosine law. Assume that the angle in a triangle is denoted by β; then, we calculate β as follows:

(46) $$\beta =\left\{\begin{array}{c} \pi \;\;\;\;\;\;\;\;\;\;\;t<-1\\ \;\arccos\;t\;\;-1\leq t\leq 1\\ 0\;\;\;\;\;\;\;\;\;\;\;t>1 \end{array}\right..$$

Above, tis calculated from the trilateral relationship according to the cosine theorem. We call the respective methods the “angle-based null space algorithm” (A-NLS) and “cosine law-based null space algorithm” (C-NLS), whereby the angles are obtained by angle measurement or the cosine law calculation. The main steps of the algorithm are shown in Algorithm 1.

**Algorithm 1:** A-NLS and C-NLS algorithms. **Input:**
$s_{i},\rho_{i},1\leq i\leq L,\;\;i\in N^{+}$ and $\alpha_{i}$ (the angle observation is unnecessary for C-NLS); **Output:**
$\hat{x}$ 1: Measuring or calculating Ω that entails errors; 2: Angle estimation is performed by using the Equation (45); 3: Obtain $\Omega^{\prime }$; 4: Calculate the null space of $\Omega^{\prime }$, and choose the appropriate $k_{i}$ so that ν≪0; 5: For ∀i, calculate $\rho_{i}^{\prime }=\rho_{i}-\nu_{i}$, set K>0, t=0, and choose random $x^{0}$; 6: **while**
$\|x^{t+1}-x^{t}\|>K$
**do** 7:  Update $x^{t+1}$ for using the Gauss–Newton iteration method; 8:  t=t+1; 9: **end while** 10: **return**
$\hat{x}=x^{t}$

## 5. Simulations and Results

In the fifth part of this paper, we will validate the proposition and the proposed algorithm using a numerical simulation. The following simulation chooses two typical scenarios of non-cooperative and cooperative positioning for analysis and verification.

### 5.1. Simulation Scenario Setting

In the noncooperative scenario, we assumed that there were four anchors and one target. In the cooperative scenario, there were two targets and six anchors. The Cartesian coordinate system was established in the two-dimensional plane. The coordinates of each anchor are shown in Table 1. Three kinds of environments were simulated: the NLOS environment, the LOS environment, and the case of negative ranging errors. We can consider the latter case as a particular condition. Although it is not common in actual positioning practice, we can regard it as the equivalent ranging error after using Proposition 3. The magnitudes of errors of each scenario in various situations are shown in Table 2 and Table 3.

### 5.2. Convexity Verification

**Figure 2:**
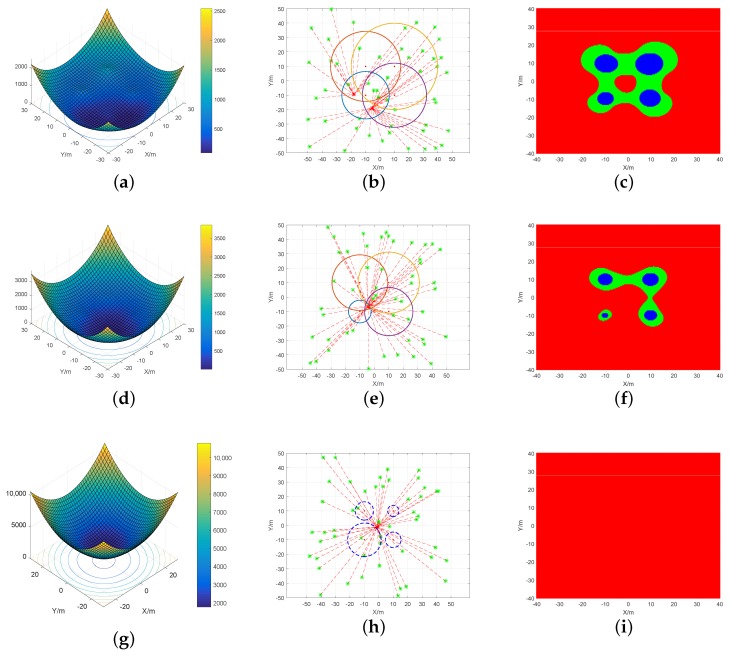
The graphs related to $F_{s}$ of one target in the noncooperative scenario. Above, Graphs (**a**,**d**,**g**) are the images of $F_{s}$ in each scene; Graphs (**b**,**e**,**h**) are the results of the Gauss–Newton iteration algorithm when different initial values were selected; Graphs (**c**,**f**,**i**) represent the semipositive definite distribution of Hessian matrix J in the two-dimensional plane.

First, function $F_{s}$ was simulated globally to observe the convexity in various environments. The convexity of $F_{s}$ was considered under various ranging errors. The function image, the semipositive definite condition, and the estimates calculated by the iterative algorithm were obtained. The results are shown in Figure 2.

Figure 2c,f,g shows the condition of Hessian matrix J at each point in the plane; the red part represents semipositive definite, the blue part seminegative definite, and the green part indefinite. Theorem 1 shows that if J is semipositive definite, the function is convex. It is observed from Figure 2c that if the targets were in the NLOS environment, the semipositive definite area was discontinuous. The sum of the indefinite and seminegative definite areas was larger. If the targets were in the LOS environment, the semipositive definite area was continuous, while the sum of indefinite and seminegative definite areas was smaller. Proposition 2 shows that if there is a positive ranging error, the cost function is nonconvex in the global range. Therefore, in the above two environments, $F_{s}$ cannot be nonconvex in the global range.

The target position was solved for by the Gauss–Newton iteration method, with different initial values selected from different directions. The results are shown in Figure 2b,e,h. In the NLOS environment, because of the positive errors, the LS cost function $F_{s}$ had more than one local optimal point due to nonconvexity. When the initial value varied, the iterative algorithm converged to different minimums. In the LOS environment, there were lower ranging errors. Although the LS cost function $F_{s}$ was also nonconvex in the global range, there was no increase in the number of local optimal points. In this case, the iterative algorithm converged to the same location. Hence, the original LS model was applicable in the LOS environment.

The results obtained if the ranging error was negative and satisfied the condition for the cost function $F_{s}$ to be convex are shown in Figure 2g,i. It is observed that J was semipositive definite and $F_{s}$ was convex in the global range. Figure 2h shows the results of the Gauss–Newton iteration algorithm with various initial values. The circle formed by the dotted lines in the graph indicates that the ranging error was negative, and its size is the absolute value of the distance observation. According to the results, the algorithm can eventually iterate to the same location regardless of the initial value, which confirms the global convergence in this case.

**Figure 3:**
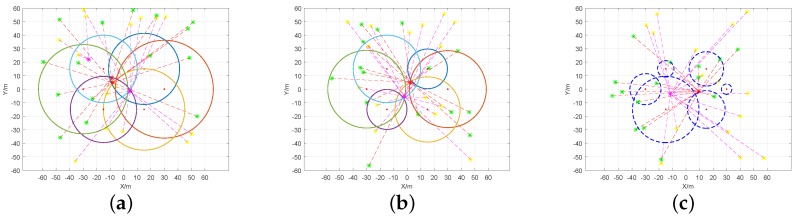
The results of the Gauss–Newton iteration algorithm when different initial values were selected in the cooperative scenario. Above, graph (**a**) shows the result for NLOS positioning environment, graph (**b**) shows the result for LOS environment, and graph (**c**) shows the result when the ranging error was negative.

In the cooperative scenario, various initial values were selected, and the target location was calculated by the Gauss–Newton iterative algorithm. The iterative images are shown in Figure 3a–c. In the NLOS positioning environment, because of the positive ranging errors, the cost function was not convex in the global range. Therefore, various initial values caused the iteration algorithm to converge to different minimums. In the LOS positioning environment, although $F_{c}$ was also nonconvex in the global range, the positive ranging error was lower; hence, the algorithm could still converge to the same local optimal point. If the ranging error was negative, the cost function was convex in the global range. Thus, regardless of the selected initial value, the algorithm would converge to the global optimal point.

### 5.3. Null Space Algorithm Performance

To compare the performance of algorithms (LS, A-NLS, and C-NLS), the following simulations were performed in both the noncooperative and cooperative scenarios. We assumed that all the targets were located in the polygon composed of adjacent nodes. The coordinates of each anchor are shown in Table 1. We performed simulations in both the LOS and NLOS environments. The ranging error parameters were set to $\mu_{nlos}=5$ m, $\sigma_{los}^{2}$ = 3 m^2^, and $\sigma_{nlos}^{2}$ = 4.5 m^2^, and in the AOA angle measurement, it was assumed that the parameters of the relative angle error matrix were $\mu_{\alpha }=0.1,\;\sigma_{\alpha }^{2}=0.5$. In the noncooperative scenario, the real location of the target was $x_{1}={\left[2,4\right]}^{T}$, while in the cooperative scenario, the real locations of the two targets were $x_{1}={\left[4,3\right]}^{T}$ and $x_{2}={\left[-6,-5\right]}^{T}$. For the LS, C-NLS, and A-NLS algorithms, any position was selected as the initial iteration value of the algorithm in each calculation. To consider all directions of the location of the initial value, the distribution ${\hat{x}}_{0}∼N\left(\mu_{0},\Sigma_{0}\right)$ was used, where $\mu_{0}$ is the mean vector and $\Sigma_{0}$ is the covariance matrix; their values were set to $\mu_{0}={\left[50,50\right]}^{T}$ and $\Sigma_{0}=\left[\begin{array}{cc} 100 & 0\\ 0 & 100 \end{array}\right]$. As a reference, we chose the SDP and PPM algorithms to compare the performance with that of the null space algorithm proposed in this paper. For the PPM algorithm, the initial iteration position of each target was equal to the average of coordinates of its adjacent nodes. For each scenario, 100 numerical simulations were performed. The estimated positions obtained by the algorithms were compared with the real positions of the targets. The root mean squared errors (RMSE) were calculated according to Formula (47).

(47) $$e_{RMSE}=\sqrt{\frac{1}{M}\sum_{j=1}^{M}{∥{\hat{x}}_{j}-x_{j}∥}_{2}}.$$

**Figure 4:**
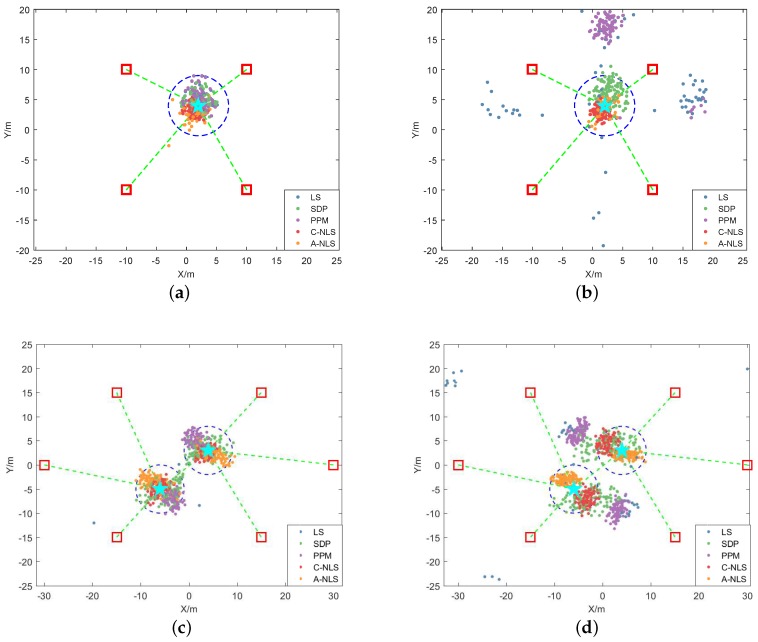
Convergence for algorithms in the different scenarios and environments. Above, Graphs (**a**,**b**) are the results in the noncooperative scenario, where (a) represents the LOS environment, while (b) represents the NLOS environment. Graphs (**c**,**d**) are the results in the cooperative scenario, which represent the LOS and NLOS environments, respectively. The red boxes in the figure represent the anchors, while the blue stars represent the targets. We drew the ranging link as the green dotted line and the convergence radius as the blue dotted circle. The solid points represent the results of positioning.

We calculated the convergence probability for different algorithms. The simulation results are shown in Figure 4, Table 4 and Table 5. If the convergence threshold was 5 m, we can see from the graphs or tables that in the LOS environment, there was a high convergence probability of each algorithm in the noncooperative scenario, while in the cooperative scenario, the convergence probability of the LS and PPM algorithm became lower. In the NLOS environment, the convergence probability of the LS and PPM algorithm decreased seriously, especially in the cooperative scenarios, which was almost non-convergent. The convergence performance of the SDP algorithm in the cooperative scenario also decreased considerably. However, the C-NLS algorithm maintained a high convergence probability in all environments and scenarios. The performance of the A-NLS algorithm was similar to that of the C-NLS algorithm, but it slightly decreased in the NLOS environment of the cooperative scenario.

**Figure 5:**
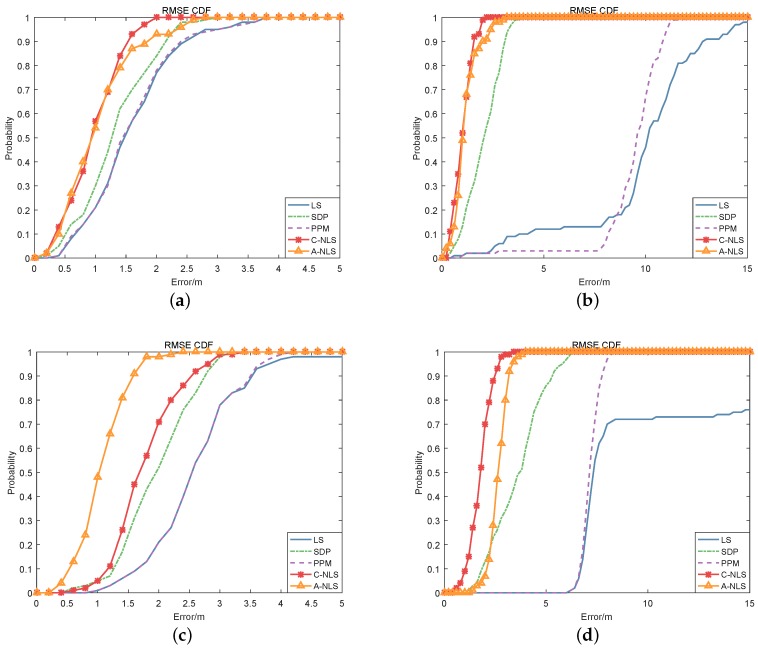
The CDF of the minimum mean squared error (MESE) for algorithms in the different scenarios and environments. Above, Graphs (**a**,**b**) are the results in the noncooperative scenario, where (a) represents the LOS environment, while (b) represents the NLOS environment. Graphs (**c**,**d**) are the results in the cooperative scenario, which represent the LOS and NLOS environments, respectively.

Afterwards, the corresponding cumulative probability distributions of errors were obtained. The results are shown in Figure 5. In particular, Figure 5a,c shows that in the LOS environment, the differences between the algorithms were not significant in both the noncooperative and cooperative scenarios. In the NLOS environment, as shown in Figure 5b,d, the differences between the algorithms were apparent. The LS and PPM algorithms showed large positioning errors in the cooperative and noncooperative scenarios. The main reason is that the positive ranging errors increased, which directly led to a sharp decline of positioning performance.

The location precision of the SDP and null space algorithms (A-NLS, C-NLS) did not decrease in the NLOS environment, which reflects the stability of these algorithms in various situations; i.e., the algorithms can obtain better location estimates in both the LOS and NLOS environments. At the same time, the null space algorithm proposed in this paper was slightly better than the traditional SDP algorithm in both noncooperative and cooperative scenarios and achieved the desired goal.

## 6. Conclusions

In this paper, a necessary and sufficient condition for the global convexity of the LS cost function was specified for WSN positioning. Generally, when all the ranging errors were far less than zero, the LS cost function was convex. Next, we defined the relative angle matrix in both the noncooperative and cooperative scenarios and proved the two essential properties. We observed that if all the targets were located in the convex polygon formed by their adjacent nodes, the LS cost function could be transformed into a globally convex function by constructing measurements with a negative distance. Based on the analysis, we proposed a robust algorithm for WSN localization. The proposed method reduced the sensitivity of the Gauss–Newton iteration algorithm to initial value selection and made the function globally convex. In other words, the function had the characteristic of large-scale convergence. In the fifth part of the article, numerical simulations were performed to verify the proposition described in the third part, and the robust algorithm was compared with the conventional methods. The results showed that the null space algorithm effectively constrained the error in the NLOS environment and obtained more accurate positioning results in both the LOS and NLOS environments.

In the future, we will carry out a study on the impact of varying the topologies and number of anchors and targets. Furthermore, we will deal with the problem when the targets are not located inside the convex polygon formed by its neighboring nodes.

## Figures and Tables

**Table 1:** Coordinates of the anchors.

Node Number	$A_{1}$	$A_{2}$	$A_{3}$	$A_{4}$	$A_{5}$	$A_{6}$
**Noncooperative/(m)**	$\left(-10,-10\right)$	$\left(-10,10\right)$	$\left(10,-10\right)$	$\left(10,10\right)$	–	–
**Cooperative/(m)**	$\left(15,15\right)$	$\left(30,0\right)$	$\left(15,-15\right)$	$\left(-15,-15\right)$	$\left(-30,0\right)$	$\left(-15,15\right)$

**Table 2:** Ranging errors in the noncooperative scenario.

Link Number	$l_{1}$	$l_{2}$	$l_{3}$	$l_{4}$
**NLOS** $\varepsilon_{i}$ **/(m)**	10.59	6.53	7.23	6.93
**LOS** $\varepsilon_{i}$ **/(m)**	2.03	1.58	−1.48	1.42
**Negative** $\varepsilon_{i}$ **/(m)**	−17.42	−24.06	−26.57	−20.69

**Table 3:** Ranging errors in the cooperative scenario.

Link Number	$l_{1}$	$l_{2}$	$l_{3}$	$l_{4}$	$l_{5}$	$l_{6}$	$l_{7}$
**NLOS** $\varepsilon_{i}$ **/(m)**	10.31	8.98	9.54	11.04	8.42	2.72	3.67
**LOS** $\varepsilon_{i}$ **/(m)**	−1.62	2.26	3.08	1.32	4.22	2.13	−2.74
**Negative** $\varepsilon_{i}$ **/(m)**	−29.13	−30.32	−34.61	−38.75	−35.69	−28.02	−36.64

**Table 4:** Convergence probability in the noncooperative scenario. PPM, parallel projection method; SDP, semidefinite programming; A-NLS, angle-based null space algorithm; C-NLS, cosine law-based null space algorithm.

Algorithms	LS	PPM	SDP	A-NLS	C-NLS
**LOS/**%	99	99	100	99	100
**NLOS/**%	6	3	94	100	100

**Table 5:** Convergence probability in the cooperative scenario.

Algorithms	LS	PPM	SDP	A-NLS	C-NLS
**LOS/**%	66	68	93	100	99
**NLOS/**%	0	0	30	78	97

**Figure A1:**
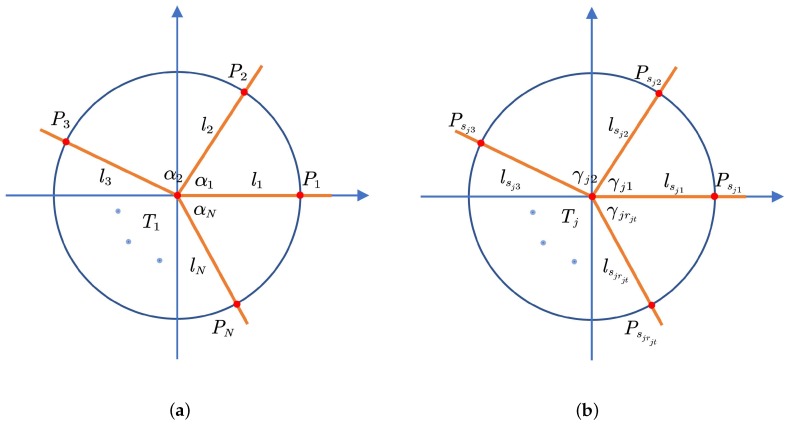
Establish the coordinate system with the user node as the origin. Draw the unit circle with the origin as the center. The points and angles defined by each link are as shown. Graph (**a**) denotes one target $T_{1}$ in the noncooperative scenario, and Graph (**b**) denotes the cooperative scenario, where one of the user nodes is $T_{j}$.

## Appendix A

Before proving the proposition, we first give the following lemma:

**Lemma** **A1.**
*A matrix, which is the sum of finite semipositive definite matrices, is still semipositive definite. That is, if $R_{i}⪰0,\;1\leq i\leq N,\;\;i\in N^{+}$, there exists $W=\sum_{i=1}^{N}R_{i}⪰0$.*

**Proof of Proposition** **1.** Consider decomposing J and the representation $J=\sum_{i=1}^{N}Q_{i}$, where:

(A1) $$Q_{i}=\left[\begin{array}{cc} \frac{{\hat{d}}_{i}-\rho_{i}}{{\hat{d}}_{i}}+\frac{\rho_{i}}{{\hat{d}}_{i}^{3}}{\left(x_{s_{i}}-{\hat{x}}_{1}\right)}^{2} & \frac{\rho_{i}}{{\hat{d}}_{i}^{3}}\left(x_{s_{i}}-{\hat{x}}_{1}\right)\left(y_{s_{i}}-{\hat{y}}_{1}\right)\\ \frac{\rho_{i}}{{\hat{d}}_{i}^{3}}\left(x_{s_{i}}-{\hat{x}}_{1}\right)\left(y_{s_{i}}-{\hat{y}}_{1}\right) & \frac{{\hat{d}}_{i}-\rho_{i}}{{\hat{d}}_{i}}+\frac{\rho_{i}}{{\hat{d}}_{i}^{3}}{\left(y_{s_{i}}-{\hat{y}}_{1}\right)}^{2} \end{array}\right].$$

To find the eigenvalues of $Q_{i}$, let:

(A2) $$\left|\lambda I-Q_{i}\right|=0.$$

Simplifying it, we obtain:

(A3) $$\lambda^{2}-\frac{2{\hat{d}}_{i}-\rho_{i}}{{\hat{d}}_{i}}\lambda +\frac{{\hat{d}}_{i}-\rho_{i}}{{\hat{d}}_{i}}=0.$$

The two roots of λ can be obtained as follows:

(A4) $$\left\{\begin{array}{c} \lambda_{1}=1\\ \lambda_{2}=\frac{{\hat{d}}_{i}-\rho_{i}}{{\hat{d}}_{i}} \end{array}\right..$$

As Formula (A4) shows, matrix $Q_{i}$ has two eigenvectors, and $\lambda_{1}>0$ is invariable. If another eigenvector satisfies $\lambda_{2}={\hat{d}}_{i}-\rho_{i}\geq 0$, then $Q_{i}⪰0$. From Lemma A1, we know that if ${\hat{d}}_{i}-\rho_{i}\geq 0,\;1\leq i\leq N,\;\;i\in N^{+}$ holds, then J⪰0. Then, the set of all estimates ${\hat{x}}_{1}$ that satisfy this condition is C. □

## Appendix B

**Proof of Property** **1.** We first show that this property holds in the noncooperative scenario with one target. Let the angles between two adjacent anchor nodes and the target be $\alpha_{1},\alpha_{2},\ldots ,\alpha_{N}$. Then, we can compute that:

$$\Omega_{s}=\left[\begin{array}{ccccc} 1 & \cos\alpha_{1} & \cos\left(\alpha_{1}+\alpha_{2}\right) & \ldots & \cos\left(\sum_{i=1}^{N-1}\alpha_{i}\right)\\ \cos\left(\sum_{i=2}^{N}\alpha_{i}\right) & 1 & \cos\alpha_{2} & \ldots & \cos\left(\sum_{i=2}^{N-1}\alpha_{i}\right)\\ \cos\left(\sum_{i=3}^{N}\alpha_{i}\right) & \cos\left(\sum_{i=3}^{N}\alpha_{i}+\alpha_{1}\right) & 1 & \ldots & \cos\left(\sum_{i=3}^{N-1}\alpha_{i}\right)\\ \vdots & \vdots & \vdots & \ddots & \vdots \\ \cos\alpha_{N} & \cos\left(\alpha_{N}+\alpha_{1}\right) & \cos\left(\alpha_{N}+\alpha_{1}+\alpha_{2}\right) & \ldots & 1 \end{array}\right]$$

(A5) $$=\left[\begin{array}{ccccc} 1 & \cos\alpha_{1} & \cos\left(\alpha_{1}+\alpha_{2}\right) & \ldots & \cos\left(\sum_{i=1}^{N-1}\alpha_{i}\right)\\ \cos\left(-\alpha_{1}\right) & 1 & \cos\left(-\alpha_{1}+\alpha_{1}+\alpha_{2}\right) & \ldots & \cos\left(-\alpha_{1}\sum_{i=1}^{N-1}\alpha_{i}\right)\\ \cos\left(-\alpha_{1}-\alpha_{2}\right) & \cos\left(-\alpha_{1}-\alpha_{2}+\alpha_{1}\right) & 1 & \ldots & \cos\left(-\alpha_{1}-\alpha_{2}-\sum_{i=1}^{N-1}\alpha_{i}\right)\\ \vdots & \vdots & \vdots & \ddots & \vdots \\ \cos\left(-\sum_{i=1}^{N-1}\alpha_{i}\right) & \cos\left(\sum_{i=1}^{N-1}\alpha_{i}+\alpha_{1}\right) & \cos\left(\sum_{i=1}^{N-1}\alpha_{i}+\alpha_{1}+\alpha_{2}\right) & \ldots & 1 \end{array}\right].$$

An elementary row transformation is performed on $\Omega_{s}$; then, the simplified matrix can be structured as:

(A6) $$\Omega_{s}^{\prime }=\left[\begin{array}{ccccc} 1 & \cos\alpha_{1} & \cos\left(\alpha_{1}+\alpha_{2}\right) & \ldots & \cos\left(\sum_{i=1}^{N-1}\alpha_{i}\right)\\ 0 & \sin\alpha_{1} & \sin\left(\alpha_{1}+\alpha_{2}\right) & \ldots & \sin\left(\sum_{i=1}^{N-1}\alpha_{i}\right)\\ 0 & 0 & 0 & \ldots & 0\\ \vdots & \vdots & \vdots & \ddots & \vdots \\ 0 & 0 & 0 & \ldots & 0 \end{array}\right].$$

From this, we observe that the rank of $\Omega_{s}$ with one target satisfies r≤2. Consider the case of more than one target in the noncooperative scenario. For each node, we can perform a similar elementary row transformation, and the number of nonzero rows is no more than two. Hence, we know that the number of nonzero rows of $\Omega_{s}^{\prime }$ will not be greater than τ. In the cooperative scenario, the first to the Nth rows of $\Omega_{c}$ are similar to those of $\Omega_{s}$. For rows numbered from N+1 to *L*, due to the existence of the TT link, the number of nonzero elements per row is four; if i=j, we have $\omega_{ij}=2$. The reason is that the targets connect between the cooperative link. If each target has at least two known location nodes connected to it, the element in the respective row can be reduced to zero by a row transformation similar to that in the noncooperative scenario. In the simplified relative angular matrix $\Omega_{c}^{\prime }$, each user node is independent of other nodes, so it must hold that r≤τ. □

## Appendix C

**Definition** **A1.**
*An adjacent node of $T_{j}$ is defined as the node that has a ranging link with $T_{j}$, whether it is an anchor or a target.*

**Definition** **A2.**
*A point p in a convex set K is said to be an extreme point if it cannot be written in the form $p=tx+\left(1-t\right)y$, where x and y are distinct points of K and 0<t<1; informally, this means p is not between two other points of K.*

**Lemma** **A2.**
*Every point in a bounded closed convex set must be a convex combination of its extreme points [40].*

**Lemma** **A3.**
*Let $K_{1},K_{2}$ be convex subsets of $R^{n}$ and $R^{m}$, respectively. Then, $K_{1}\times K_{2}\subset R^{n}\times R^{m}R^{m+n}$ is convex. Furthermore, point $\left(P_{1},P_{2}\right)$ is an extreme point of $K_{1}\times K_{2}$ if and only if $P_{1}$ is an extreme point of $K_{1}$ and $P_{2}$ is an extreme point of $K_{2}$ [40].*

**Proof of Property** **2.** We first assume that there is only one node in the scenario, and the error vector is set to $\varepsilon ={\left[\varepsilon_{1}\;\varepsilon_{2}\;\ldots \;\varepsilon_{N}\right]}^{T}$. According to Formula (32),

(A7) $$\left\{\begin{array}{c} \varepsilon_{1}+\sum_{i=2}^{N}\cos\left(\sum_{j=1}^{i-1}\alpha_{j}\right)\varepsilon_{i}=0\\ \;\;\;\;\;\sum_{i=2}^{N}\sin\left(\sum_{j=1}^{i-1}\alpha_{j}\right)\varepsilon_{i}=0 \end{array}\right..$$

As Figure A1a shows, if a coordinate system is established with $T_{1}$ as the origin, then Formula (32) can be rewritten as follows:

(A8) $$\left[\begin{array}{c} 1\\ 0 \end{array}\right]\varepsilon_{1}+\sum_{i=2}^{N}\left[\begin{array}{c} \cos\left(\sum_{j=1}^{i-1}\alpha_{j}\right)\\ \sin\left(\sum_{j=1}^{i-1}\alpha_{j}\right) \end{array}\right]\varepsilon_{i}=\left[\begin{array}{c} 0\\ 0 \end{array}\right].$$

Take $T_{1}$ as the center of a circle; consider a unit circle, and suppose that the intersection with $l_{i}$ occurs at point $P_{i}$. It is easy to observe that $\left[\begin{array}{c} 1\\ 0 \end{array}\right],\left[\begin{array}{c} \cos\alpha {\;}_{1}\\ \sin\alpha {\;}_{1} \end{array}\right],\ldots ,\left[\begin{array}{c} \cos\left(\sum_{j=1}^{N-1}\alpha_{j}\right)\\ \sin\left(\sum_{j=1}^{N-1}\alpha_{j}\right) \end{array}\right]$ are the coordinates of points $P_{1},P_{2},\ldots ,P_{N}$ in the Cartesian coordinate system. If $P_{1},P_{2},\ldots ,P_{N}$ form a convex polygon and $P_{1},P_{2},\ldots ,P_{N}$ are the extreme points of that polygon, the origin $T_{1}$ must be a point inside the convex polygon. Lemma 3 shows that there exists a convex combination, so that the coordinates of $T_{1}$ are composed of convex combinations of $P_{1},P_{2},\ldots ,P_{N}$. In other words, $\exists \;\xi_{1},\xi_{2},\ldots ,\xi_{N}$, $\xi_{i}\in \left(0,1\right)$ and $\sum_{i=1}^{N}\xi_{i}=1$ such that:

(A9) $$\left[\begin{array}{c} 1\\ 0 \end{array}\right]\xi_{1}+\sum_{i=2}^{N}\left[\begin{array}{c} \cos\left(\sum_{j=1}^{i-1}\alpha_{j}\right)\\ \sin\left(\sum_{j=1}^{i-1}\alpha_{j}\right) \end{array}\right]\xi_{i}=\left[\begin{array}{c} 0\\ 0 \end{array}\right].$$

Let $\varepsilon_{i}=\xi_{i}$; then, we can prove that Property 2 holds in the single target scenario. If more than one node exists, we suppose that the $j^{\mathrm{th}}$ user node $T_{j}$ has a total of $r_{jt}$ links, including $r_{ja}$ AT links and $r_{jc}$ TT links. These links divide the plane into $r_{jt}$ parts. Every two adjacent links form an angle with the $T_{j}$ as the vertex. Let the radian measures of angles at $T_{j}$ be $\gamma_{j1},\gamma_{j2},\ldots ,\gamma_{j\left(r_{ja}-1\right)},\gamma_{jr_{ja}},\ldots ,\gamma_{jr_{jt}}$. The two adjacent links constituting these angles are numbered $l_{s_{j1}},l_{s_{j2}},\ldots ,l_{s_{jr_{jt}}},l_{s_{j1}}$, where $s_{jk}$ is the number of all links connected to $T_{j}$, which satisfies $s_{j\left(k+1\right)}>s_{jk}$. The error vector is set to $\varepsilon ={\left[\varepsilon_{1}\;\varepsilon_{2}\;\ldots \;\varepsilon_{L}\right]}^{T}$; according to Formula (36), it holds that:

(A10) $$\left\{\begin{array}{c} \begin{array}{c} \varepsilon_{s_{11}}+\sum_{k=2}^{r_{1t}}\cos\left(\sum_{m=1}^{k-1}\gamma_{1m}\right)\varepsilon_{s_{1k}}=0\\ \begin{array}{c} \;\;\;\;\;\sum_{k=2}^{r_{1t}}\sin\left(\sum_{m=1}^{k-1}\gamma_{1m}\right)\varepsilon_{s_{1k}}=0\\ \;\;\;\;\;\;\;\;\;\;\;\;\;\;\;\;\;\;\;\;\;\;\;\;\;\;\;\;\vdots \end{array}\\ \varepsilon_{s_{M1}}+\sum_{k=2}^{r_{Mt}}\cos\left(\sum_{m=1}^{k-1}\gamma_{Mm}\right)\varepsilon_{s_{Mk}}=0\\ \;\;\;\;\;\sum_{k=2}^{r_{Mt}}\sin\left(\sum_{m=1}^{k-1}\gamma_{Mm}\right)\varepsilon_{s_{Mk}}=0 \end{array} \end{array}\right..$$

As shown in Figure A1b, consider any $T_{j}$, and consider a unit circle centered at $T_{j}$ that intersects $l_{i}$ at points $P_{s_{j1}},P_{s_{j2}},\ldots ,P_{s_{jr_{jt}}}$. Then, the jth and j+1th lines in Equation (A10) can be written as follows:

(A11) $$\left[\begin{array}{c} 1\\ 0 \end{array}\right]\varepsilon_{s_{j1}}+\sum_{k=2}^{r_{jt}}\left[\begin{array}{c} \cos\left(\sum_{m=1}^{k-1}\gamma_{jm}\right)\\ \sin\left(\sum_{m=1}^{k-1}\gamma_{jm}\right) \end{array}\right]\varepsilon_{s_{jk}}=\left[\begin{array}{c} 0\\ 0 \end{array}\right],$$

where $\left[\begin{array}{c} 1\\ 0 \end{array}\right],\left[\begin{array}{c} \cos\gamma_{j1}\\ \sin\gamma_{j1} \end{array}\right],\ldots ,\left[\begin{array}{c} \cos\left(\sum_{m=1}^{r_{jt}}\gamma_{jm}\right)\\ \sin\left(\sum_{m=1}^{r_{jt}}\gamma_{jm}\right) \end{array}\right]$ represent the coordinates of points $P_{s_{j1}},P_{s_{j2}},\ldots ,P_{s_{jr_{jt}}}$ in the Cartesian coordinate system. Then, $P_{s_{j1}},P_{s_{j2}},\ldots ,P_{s_{jr_{jt}}}$ form a convex polygon, and they are the extreme points of that polygon. Hence, all the points inside the polygon constitute a closed convex set $H_{j}\subseteq R^{2}$. If all $T_{i}$ are located in the corresponding convex polygons, then set $H_{j}$ exists for $\forall j,\;1\leq j\leq M,\;\;j\in N^{+}$. As can be observed from Lemma A3, the Cartesian product $H_{t}=H_{1}\times H_{2}\times \ldots \times H_{M}\subseteq R^{\eta }$ is also a convex set. Combining extreme points ${\hat{x}}_{j},\;1\leq j\leq M,\;\;j\in N^{+}$ in $H_{j}$ forms a new extreme point $\hat{x}={\left[{\hat{x}}_{1}\;{\hat{x}}_{2}\;\ldots \;{\hat{x}}_{M}\right]}^{T}\in R^{2M}$ in $H_{t}$ in $H_{t}$. According to Lemma A2, the origin is a convex combination of extreme points. In other words, $\exists \;\xi_{1},\xi_{2},\ldots ,\xi_{L}$, $\xi_{i}\in \left(0,1\right)$ and $\sum_{i=1}^{L}\xi_{i}=1$ satisfy Equation (37). If $\varepsilon_{i}=\xi_{i}$, then Property 2 also holds in the multitarget scenario. □
